# Visible Light-Driven Photocatalytic Activity and Kinetics of Fe-Doped TiO_2_ Prepared by a Three-Block Copolymer Templating Approach

**DOI:** 10.3390/ma14113105

**Published:** 2021-06-05

**Authors:** Antonietta Mancuso, Olga Sacco, Vincenzo Vaiano, Barbara Bonelli, Serena Esposito, Francesca Stefania Freyria, Nicola Blangetti, Diana Sannino

**Affiliations:** 1 Department of Industrial Engineering, University of Salerno, via Giovanni Paolo II, 132, 84084 Fisciano, Italy; anmancuso@unisa.it (A.M.); vvaiano@unisa.it (V.V.); 2 Department of Chemistry and Biology “A. Zambelli”, University of Salerno, via Giovanni Paolo II, 132, 84084 Fisciano, Italy; 3 Unit of Torino Politecnico, Department of Applied Science and Technology and INSTM, Politecnico di Torino, Corso Duca degli Abruzzi 24, 10129 Torino, Italy; barbara.bonelli@polito.it (B.B.); serena_esposito@polito.it (S.E.); francesca.freyria@polito.it (F.S.F.); nicola.blangetti@polito.it (N.B.)

**Keywords:** visible light irradiation, Fe-doped TiO_2_, photocatalysis, discoloration, mineralization, acid orange 7, degradation kinetics, triblock copolymer template

## Abstract

Fe-doped titania photocatalysts (with 1, 2.5, and 3.5 wt. % Fe nominal content), showing photocatalytic activity under visible light, were prepared by a soft-template assisted sol–gel approach in the presence of the triblock copolymer Pluronic P123. An undoped TiO_2_ photocatalyst was also prepared for comparison. The photocatalysts were characterized by means of X-ray powder Diffraction (XRPD), Quantitative Phase Analysis as obtained by Rietveld refinement, Diffuse Reflectance (DR) UV−Vis spectroscopy, N_2_ adsorption/desorption at −196 °C, electrophoretic mobility in water (ζ-potential), and X-ray photoelectron spectroscopy (XPS). The physico-chemical characterization showed that all the samples were 100% anatase phase and that iron was present both in the bulk and at the surface of the Fe-doped TiO_2_. Indeed, the band gap energy (Eg) decreases with the Fe content, with Tauc’s plot determined values ranging from 3.35 (undoped TiO_2_) to 2.70 eV (3.5 wt. % Fe). Notwithstanding the obtained Eg values, the photocatalytic activity results under visible light highlighted that the optimal Fe content was equal to 2.5 wt. % (Tauc’s plot determined Eg = 2.74 eV). With the optimized photocatalyst and in selected operating conditions, under visible light it was possible to achieve 90% AO7 discoloration together with a TOC removal of 40% after 180 min. The kinetic behavior of the photocatalyst was also analyzed. Moreover, the tests in the presence of three different scavengers revealed that the main reactive species are (positive) holes and superoxide species. Finally, the optimized photocatalyst was also able to degrade phenol under visible light.

## 1. Introduction

Environmental pollution has been rapidly increasing due to the growing demand for various materials deriving from chemical industry [1]. One of the critical sources of environmental contamination is wastewater pollution by dyes that can also be found in groundwater [2], like azo dyes [3]. When dyes come in wastewater, they become more stable creating complex chemical structures [4] that are difficult to biodegrade and/or detect by conventional techniques [5,6]; indeed, azo dyes are currently considered as a class of contaminants of emerging pollutants, as concern exists about their actual transport, fate, toxicity, etc. [7].

Dyes discharged into wastewater can generate dangerous by-products from oxidation, hydrolysis, etc., determining serious problems to receiving ecosystems and on human health [8,9]. In particular, their release into aquatic ecosystem could cause a reduction of light penetration and dissolved oxygen concentration [10]. For this reason, the scientific world has felt the necessity to develop sustainable technologies for the degradation or elimination of pollutants from wastewaters.

Among the various advanced oxidation processes (AOPs) based on the generation of highly reactive radical species (such as hydroxyl radicals), heterogeneous photocatalysis has attracted much attention, as it does not imply the use of strong oxidants, like ozone, for instance, nor produce toxic by-products, like chlorination [11].

Along with ZnO, titanium dioxide (TiO_2_) semiconductors are the most used photocatalyst for their intrinsic physico-chemical properties, namely, as high photo-corrosion resistance in aqueous media, high chemical stability, low toxicity, and low cost. Moreover, TiO_2_ is available in several morphologies, polymorphic compositions, and nanosized particles.

When TiO_2_ is irradiated by light energy greater than its band gap electrons are transferred from the valence band (VB) to the conduction band (CB), generating positive holes in VB [12]. The photogenerated hole–electron pairs react with water and/or dissolved oxygen molecules with the production of highly reactive radical species, which play a key role in redox reactions to eliminate water organic pollutants. The three most known polymorphs of TiO_2_ are anatase, rutile, and brookite, with average band gap values of 3.2, 3.0, and 3.4 eV, respectively. Notwithstanding the slightly larger bad gap as compared to rutile, anatase exhibits higher photocatalytic activity [13] due to low surface energy [14]: ~10–20 nm large anatase NPs can be easily obtained and, being an indirect band gap semiconductor (at variance with rutile and brookite, both direct semiconductors), the photogenerated electrons and holes have longer lifetime [15].

Unfortunately, TiO_2_ absorbs below 380 nm and is activated by UV light, being able to exploit only a small fraction (~4%) of the solar spectrum. A possible solution is to reduce the band gap and extend the light absorption towards the visible region by doping with several transition metals ions [16,17]. For instance, Fe^3+^ ions with half-filled 3*d* orbitals and an ionic radius (0.65 Å) rather similar to that of Ti^4+^ ion (0.68 Å), can extend the absorption towards the visible range [18].

When Fe-doping is effective, charge-transfer transitions occurs between *d* electrons of the transition metal and the conduction band of TiO_2_ [19]. Moreover, doping with Fe^3+^ ions reduce the recombination of the photogenerated electrons and holes and enhances the absorption in the UV range [18,20,21,22]. This is possible, however, at low doping levels, as high levels induce the formation of defects, which, in turn, favor electrons/holes recombination, with a detrimental effect on photocatalytic activity [23].

Different preparation methods can be used to obtain TiO_2_-based photocatalyst such as hydrothermal or solvothermal synthesis [24,25], sol–gel methods [26,27,28,29], impregnation [30,31], and coprecipitation [32].

Butler et al. prepared Fe-doped TiO_2_ by a conventional solid-state reaction method for Acid Orange 7 (AO7) degradation [33]. Ganesh et al. prepared Fe-doped TiO_2_ powders by a conventional co-precipitation technique for degradation of methylene blue under visible light [32]. Fe-doped TiO_2_ photocatalysts were synthesized by hydrothermal method both for degradation of Malachite Green dye [34] and for abatement of active yellow XRG dye [35] under UV and visible light irradiation. Moreover, Di Paola et al. studied the photocatalytic oxidation of some carboxylic acids and 4-nitrophenol, using the same doped photocatalyst obtained by impregnation [30,31].

The sol–gel method offers several advantages such as homogeneity at molecular level, low synthesis temperature, control over microstructure, and its ability to tune the particle size, distribution, and morphology through the reaction parameters [36,37,38,39].

TiO_2_-based materials with different shape and reactivity are obtained by taking advantage of self-assembly of the structure-directing agent, making it competitive with the commercial Evonik AEROXIDE® TiO_2_ P 25. Depending on the mechanism of gel formation, surfactants can also influence the kinetics of the whole process, in addition acting as soft structural templates [40].

Fe-doped TiO_2_ photocatalyst prepared by different sol–gel methods were tested for the degradation of several dyes and other organic pollutants under vis or UV light [16,41,42].

Template-assisted sol–gel (TASG) synthesis allows inducing mesoporosity in the final material, allowing facile diffusion of reactants and products in photocatalytic processes [43].

Amphiphilic di- or tri-block copolymers are commonly used for the preparation of mesoporous titania. The commercially available Pluronics consist of PEO-block-poly(propylene oxide)-block-PEO (PEO-PPO-PEO) that are offered with different block sizes. Tri-block copolymers form stable micelles at very low concentrations and the engineering of the pore structure of the oxide is the result of a formation mechanism, whereby the sol-gel precursor hydrolyses and condenses around the mesostructured polymer phase. The removal of the template is ensured by the subsequent thermal treatment, which also induces stiffening of the inorganic network and crystallization.

In previous studies, Fe-doped TiO_2_ samples (Fe content in the 0.8–2.5 wt.% range) were obtained by TASG method in the presence of a triblock copolymer and using Ti *t*-butoxide as precursor [44,45]. The samples were tested as catalysts in the degradation of AO7 in the presence of H_2_O_2_ under different conditions of illumination, i.e., in dark conditions, under UV light, and under simulated solar light. The sample at 2.5 wt.% Fe was effective under solar illumination even in the absence of H_2_O_2_, confirming the Fe doping led to a material able to efficiently exploit the UV fraction of the solar spectrum [46].

In the present study, we have investigated the effect of the iron content on the photocatalytic discoloration and mineralization of AO7 solution under visible light irradiation using Fe-doped TiO_2_ nanoparticles. The TASG synthesis procedure was carried out by replacing the former Ti precursor (Ti *t*-butoxide) with (less expensive) Ti-*n*-butoxide and the physico-chemical characterization was carried out by means of an integrated techniques approach, with the aim of finding possible differences with respect to previously studied samples.

## 2. Materials and Methods

All the reagents for the syntheses were ACS-grade chemicals from Sigma-Aldrich, Italy.

The TiO_2_ NPs were synthesized as follows: two solutions were prepared, solution A was obtained by dropwise adding 20.0 g Ti(OBut)_4_ (titanium *n*-butoxide, 97%) to 120.0 mL acetic acid solution (20%, *v*/*v*); solution B was obtained by mixing 12.0 g Pluronic P123 ((poly (ethylene glycol)-block-poly (propylene glycol)-block-poly (ethylene glycol)) and ~80.0 mL ethanol and both solution were kept under vigorous stirring for approximately 4 h. Solution B was then added dropwise to solution A: the resulting mixture was sealed, stirred for 24 h at room temperature, and transferred into a Teflon autoclave for hydrothermal treatment at 98 °C for 48 h. The resulting precipitate was centrifuged, dried at 80 °C, and calcined in air at 450 °C for 4 h [6,7]. The Fe-doped TiO_2_ NPs with nominal iron contents of 1, 2.5, and 3.5 wt. % were prepared by following a similar procedure after addition of proper amounts of iron(III) chloride hexahydrate, FeCl_3_·6H_2_O to solution A. The obtained powders were referred to as Fe-(x)-TiO_2_, where x stands for the nominal mass percentage of Fe (Fe(1.0)-TiO_2_; Fe(2.5)-TiO_2_; Fe(3.5)-TiO_2_), while TiO_2_ stands for the undoped mesoporous TiO_2_.

### 2.1. Physico-Chemical Characterization

The Specific Surface Area (SSA) values were measured by N_2_ physisorption at −196 °C (Quantachrome Autosorb 1C, Boyton Beach, FL, USA) on powders previously outgassed at 150 °C for 4 h to remove water and other atmospheric contaminants; the samples SSA was determined according to the Brunauer–Emmett–Teller (BET) method. Total pore volume was measured at P/P^0^ = 0.99.

The powder X-ray diffraction (XRPD) patterns were collected on an X’Pert Phillips diffractometer (PANalytical, Almelo, Netherlands) equipped with Cu Kα radiation = 1.541874 Å (10–90 2θ range; step width = 0.05 2θ; time per step = 0.2 s). XRD patterns were indexed according to the Powder Data File Database (PDF-2 2004, International Centre of Diffraction Data, Pennsylvania). The phase(s) content (Quantitative Phase Analysis (QPA)) was evaluated by the full-profile Rietveld method applied to the diffraction patterns by using the X’Pert High Score Plus 3.0e software. Crystallites’ size has been evaluated using the Williamson–Hall method [47].

Transmission electron microscopy was used to investigate the NPs morphology on a Philips CM12 (Philips, Eindhoven, Netherlands) instrument, operating at 120 kV with a LaB6 filament.

ζ-potential curves of the TiO_2_ NPs were obtained by measuring the electrophoretic mobility as a function of pH by means of electrophoretic light scattering (ELS) on a Zetasizer Nano-ZS (Malvern Instruments, Worcestershire, UK). In a typical measurement, the powder was suspended in ultrapure water (MilliQ) and either sonicated for 2 min (10 W/mL, 20 kHz, Sonoplus, Bandelin, Berlin, Germany) or magnetically stirred for 5 min. The ζ-potential was measured at r.t. after adjusting the pH gradually by addition of either 0.1 M NaOH or 0.1 M HCl.

X-ray Photoelectron Spectroscopy (XPS) analysis was carried out on an XPS PHI 5000 Versa probe apparatus (ULVAC-PHI Inc., Kanagawa, Japan), using a band-pass energy of 187.85 eV, a 45° take off angle, and a 100.0 μm diameter X-ray spot size for survey spectra.

Fourier transform (FT) IR spectra were recorded at 2 cm^−1^ resolution on a Bruker Equinox 55 spectrophotometer (Bruker Italia SrL, Milano, Italy) equipped with a mercury cadmium telluride (MCT) cryodetector. For IR measurements, samples were shaped into thin, self-supporting wafers (~2 mg cm^−2^) and pretreated in a standard vacuum frame (residual pressure below 10^−3^ mbar) at room temperature, 150 and 300 °C to remove water and other atmospheric contaminants.

Diffuse Reflectance (DR) UV/Vis spectra of powder samples were recorded by UV−Vis Varian Cary 5000 spectrophotometer (Varian Instruments, Mulgrave, Australia) equipped with an integration sphere for the DR measurements.

### 2.2. Photocatalytic Activity Tests

Photocatalytic activity tests were carried out on 100 mL of AO7 aqueous solution (initial concentration = 10 ppm and pH = 5.95) in a cylindrical batch photoreactor in Pyrex (ID = 2.6 cm, L_TOT_ = 41 cm and V_TOT_ = 200 mL). A strip of visible-LEDs was positioned around the external body of the photoreactor to uniformly and intensely irradiate the volume of the solution through the transparent geometrical photoreactor surface. The photocatalyst powders were added to the AO7 aqueous solution and the suspension was maintained in dark conditions for 120 min to reach the adsorption/desorption equilibrium of the contaminant on the photocatalyst surface.

The photocatalytic test was then started under visible light irradiation up to 180 min. During the light irradiation, the reaction suspension was continuously mixed using a magnetic stirrer to avoid the sedimentation of the photocatalyst at the bottom of the photoreactor. The photocatalytic reactor was equipped with an air distributor device (Q_air_ = 150 cm^3^·min^−1^ (STP)) to assure the presence of oxygen in the reaction medium. In addition, a fan cooling system was positioned near the photoreactor to prevent an increase of the reaction temperature with the lighting of the LEDs. Table 1 reports the reaction conditions used for the photocatalytic tests.

Approximately 3 mL of the suspension was taken from the photoreactor at different times and suitably centrifuged to remove the catalyst particles from the liquid phase. The aqueous supernatant solution was then analyzed by a UV–Vis spectrophotometer (Evolution 201, Thermo Scientific, Italy, ) to assess the reaction progress. In detail, the color removal of the AO7 dye was monitored by measuring the maximum absorbance value at 485 nm. The level of mineralization was determined by measuring the total organic carbon (TOC) content of the treated solutions. The TOC of the solution was measured from CO_2_ obtained by the high temperature (680 °C) catalytic combustion method [48]. The amount of AO7 dye adsorbed on the powder surface was calculated by following equation:

(1) $$\frac{m_{AO7adsorbed}}{m_{catalyst}}=\frac{\left(Co-C\right)\times V}{m_{catalyst}}$$

where *C* = pollutant concentration after 120 min in dark (mg L^−1^), *C*_0_ = initial pollutant concentration (mg L^−1^), *V* = volume of treated solution, and *m_catalyst_* = mass of photocatalyst added in the test.

The discoloration and mineralization efficiency were calculated according to the following relationships:

(2) $$Discoloration\;efficiency=\left(1-\frac{C}{C_{0}}\right)\times 100$$

(3) $$TOC\;removal\;\left(mineralization\right)=\left(1-\frac{TOC}{TOC_{0}}\right)\times 100$$

where *C* = pollutant concentration at the generic irradiation time (mg L^−1^), *C*_0_ = initial pollutant concentration (mg L^−1^), *TOC* = total organic carbon at the generic irradiation time (mg L^−1^), and *TOC*_0_ = initial total organic carbon (mg L^−1^).

An additional photocatalytic test for the degradation of phenol (at 10 mg L^−1^ initial concentration) was carried out on the catalytic formulation that showed the best performances in the AO7 discoloration and mineralization. Phenol residual concentration was monitored by measuring its maximum absorption at 270 nm [49] by using an UV–Vis spectrophotometer (Evolution 201, Thermo Scientific, Italy).

## 3. Results

### 3.1. Physico-Chemical Characterization

**Figure 1:**
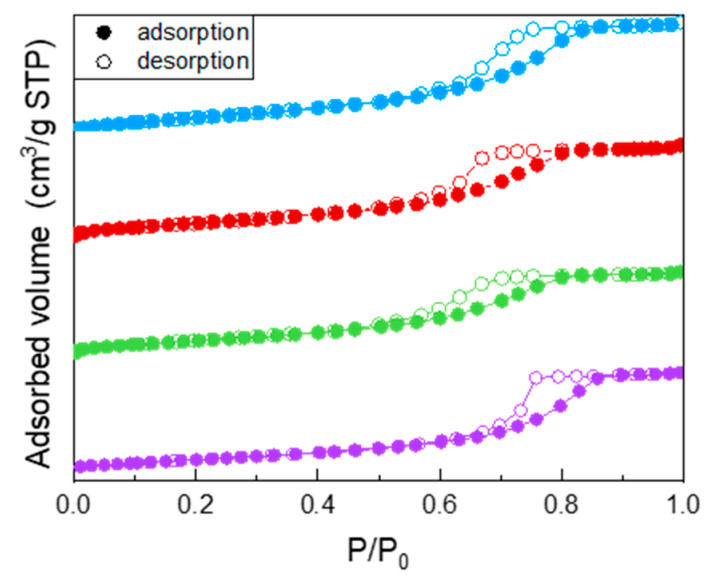
N_2_ adsorption/desorption isotherms at −196 °C obtained with the following samples: undoped TiO_2_ (purple circles), Fe(3.5)-TiO_2_ (green circles), Fe(2.5)-TiO_2_ (red circles), Fe(1.0)-TiO_2_ (light blue circles).

All the samples showed a type IV isotherm of N_2_ adsorption/desorption at −196 °C (Figure 1), ascribed to the occurrence of multi-layer adsorption and capillary condensation within both intra- and inter-particle mesopores. Specifically, the undoped TiO_2_ isotherm (purple circles) showed a H2 type hysteresis loop, typical of inkbottle mesopores. Doping with Fe leads to a change in the shape of the hysteresis loop, indicating the likely occurrence of slit-pores, and to a decrease of the values of both BET SSA and porous volume (reported in Table 2): such decrease was, however, limited and not linearly proportional to the Fe nominal content, indicating that Fe probably induced some disorder in the system, but not with a linear dependence on its (small) nominal content. As a whole, Fe doping had a limited effect on the samples BET SSA and pore volume, in agreement with the low amount of dopant.

**Figure 2:**
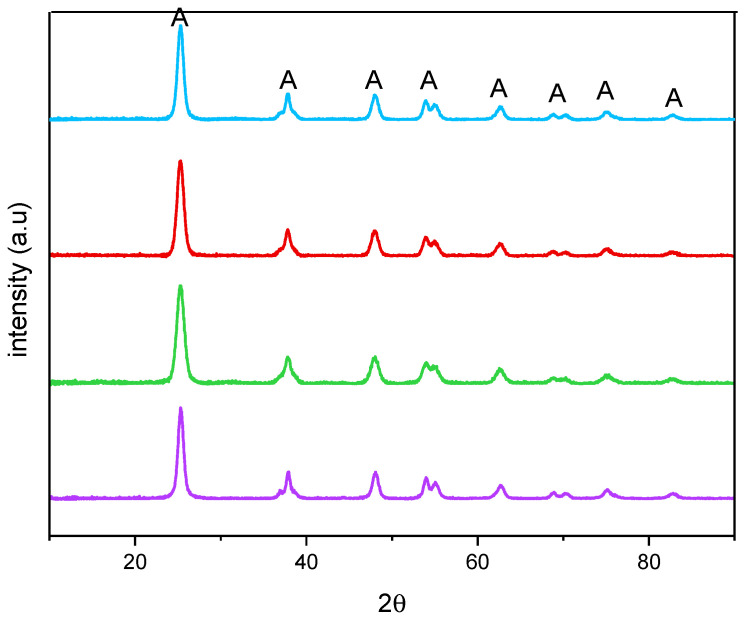
X-ray Powder Diffraction patterns obtained with the undoped TiO_2_ (purple), Fe(3.5)-TiO_2_ (green), Fe(2.5)-TiO_2_ (red), and Fe(1.0)-TiO_2_ (light blue).

Figure 2 reports the XRPD patterns of the studied samples. All the samples showed only anatase-related (A) peaks and, accordingly, an anatase content of 100 wt. % was determined by QPA, showing that no segregation of Fe-containing phases occurred. Additionally, the presence of Fe did not bring about any significant shift of the peaks of anatase, with the undoped TiO_2_ sample, at 25.5 (101), 37.4 (004), 47.9 (200), 54.0 (105), 54.9 (211), 62.6 (204), 68.9 (116), and 82.5 (224) 2θ values (PDF-2 card number 01-084-1285, released in 2004). Doping with Fe, instead, induced a change in the crystallite size, as obtained by applying the Williamson–Hall method, the corresponding values being reported in Table 2 along with the related error bars. As a whole, Fe doping leads to an overall decrease in the crystallite size with all the studied samples, although it was not possible to find a linear correlation with the nominal Fe content, likely due to the fact that Fe-doping affects both the bulk and the surface of the materials, as determined by XPS analysis (vide infra).

**Figure 3:**
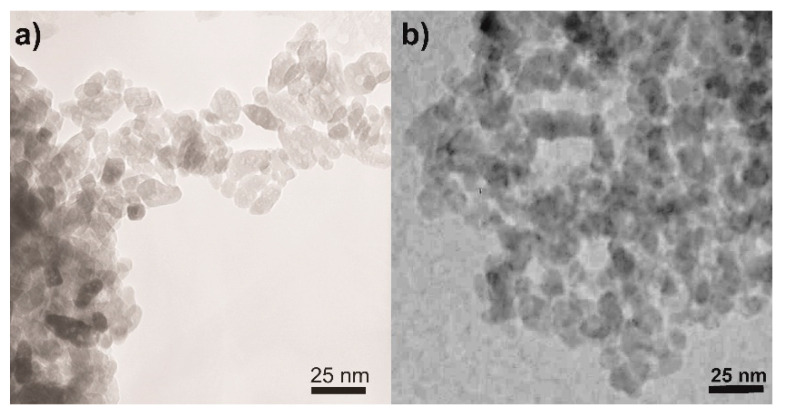
Selected TEM micrographs as obtained with the (**a**) undoped TiO_2_ and (**b**) the Fe(2.5)-TiO_2_ sample.

Concerning the NPs morphology, Figure 3 shows the TEM micrographs of two selected samples, namely, the undoped TiO_2_ (Figure 3a) and the sample at the intermediate Fe content, i.e., Fe(2.5)-TiO_2_ (Figure 3b): in agreement with previous studies showing that this synthesis leads to the formation of elongated particles with rather uniform shape and dimension, forming agglomerates with interparticle mesoporosity [45,51] and that Fe-doping does not alter the NPs shape [45,51], the TEM analysis showed the occurrence of agglomerated NPs of rather uniform shape and size with both samples.

**Figure 4:**
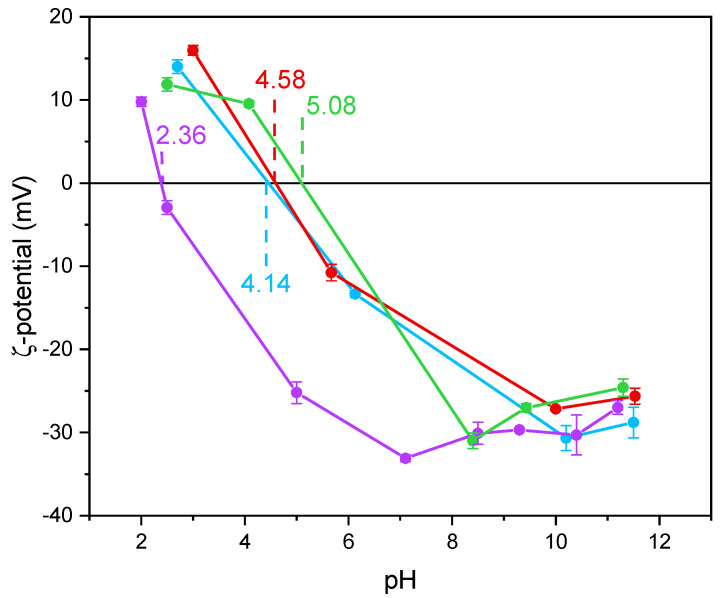
ζ-potential curves obtained by measuring the electrophoretic mobility as a function of pH obtained with the undoped TiO_2_ (purple line), Fe(3.5)-TiO_2_ (green line), Fe(2.5)-TiO_2_ (red line), Fe(1.0)-TiO_2_ (light blue line). For each point, an average of three measures is reported, with the related error bar.

Notoriously, Ti-OH groups at the surface of TiO_2_ have an amphoteric behavior, being protonated and deprotonated below and above the pH_IEP_, respectively. Such a behavior may affect the interaction with species in solution, like in this work. The values of pH_IEP_ as obtained by the ζ-potential measurements in Figure 4 and reported in Table 2 show that the undoped TiO_2_ sample had a particularly low value, as compared to literature values usually reported for P25 NPs with average size in the 20 to 40 nm range [52] and for pure anatase NPs with average size in the 7.0 to 20 nm range [53]. According to Suttiponparnit et al. [53], both primary particle size and surface area affect the charge of TiO_2_ NPs obtained by sol–gel methods, whereas the type of crystalline phase should not. In contrast, other authors [54] measured different pH_IEP_ with brookite, anatase, and rutile samples obtained by hydrothermal treatment (in the order pH_IEP_ brookite < pH_IEP_ anatase < pH_IEP_ rutile), with brookite showing stronger Brønsted sites; the same authors also measured slightly lower pH_IEP_ with more crystallized solids. With a set of undoped TiO_2_ samples of different phase composition, we measured low pH_IEP_ values with brookite-containing samples [44]. Other authors pointed out that the type of synthesis plays a prominent role on the TiO_2_ surface properties [55,56].

Holmberg et al. [52] predicted that TiO_2_ NPs with a diameter below 10 nm have a higher surface charge with respect to larger NPs and, thus, the pH_IEP_ should increase as the NPs size decreases. As a whole, however, very different pH_IEP_ values are reported in the literature for TiO_2_ samples and very different explanations are provided (particle size, synthesis method, type of polymorph, etc.). Here, we notice that doping with Fe led to a change in the pH_IEP_ (Figure 4), indicating the occurrence of Fe species also at the NPs surface and not only in the bulk. Indeed, in a previous paper, a similar synthesis procedure led to the presence of Fe species both at the surface and in the bulk of Fe-doped TiO_2_ [46]. Here, XPS was used to measure the Fe/Ti atomic ratio at the surface, to be compared to the nominal Fe/Ti atomic ratio. The corresponding values, reported in Table 2, showed an iron-enriched surface of the studied samples, which we ascribe to the synthesis procedure. Accordingly, the measured pH_IEP_ increases with the Fe content, in agreement with the more basic character of Fe_2_O_3_ with respect to TiO_2_ and with the hypothesis of a certain Fe concentration at the samples surface [57].

**Figure 5:**
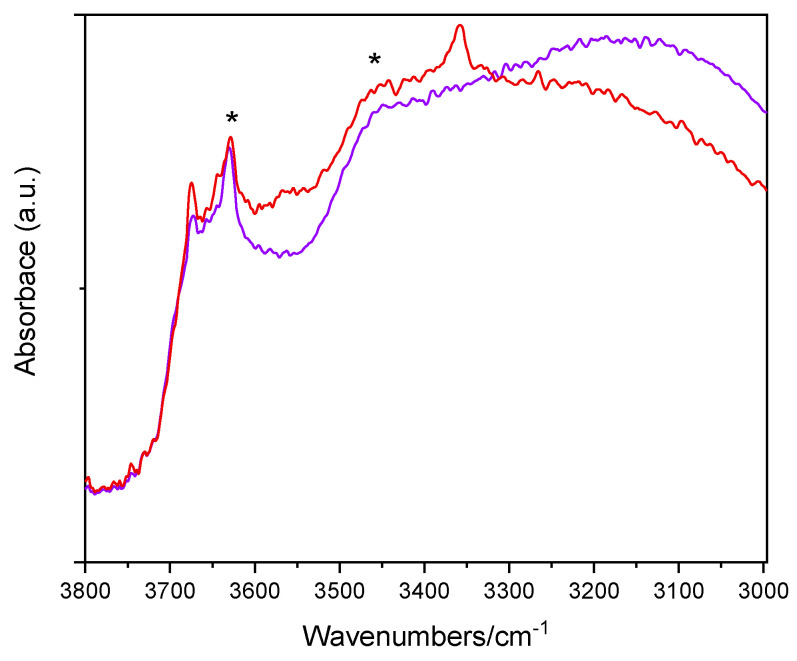
Selected IR spectra obtained with the undoped TiO_2_ sample (purple) and the Fe(2.5)-TiO_2_ sample (red). The bands at 3630 and ~3460 cm^−1^ are labelled by asterisks (*).

Figure 5 reports the IR spectra (OH stretching range: 3800–3000 cm^−1^) of two selected samples, namely, the undoped TiO_2_ sample and the Fe(2.5)-TiO_2_ sample (at intermediate Fe content) after prolonged evacuation at room temperature, in order to remove weakly physisorbed water molecules, without further perturbing the surface. The lower wavenumber region shows, instead, a band envelope, due to carbonates and bicarbonates species (not shown). As expected, the OH spectra are dominated by the IR features of the H-bond, due to the presence of adsorbed water: in particular, the bands at 3630 cm^−1^ and ~3460 cm^−1^ (asterisks) are, respectively, ascribed to the asymmetric and symmetric OH stretching modes of H_2_O molecules strongly adsorbed on Ti^4+^ sites, the corresponding bending mode being observed at 1620 cm^−1^ (not shown). The bands at 3674 and 3644 cm^−1^ are ascribed to different types of Ti-OH species, usually observed at the surface of anatase crystalline planes [58]. Interestingly, with the Fe(2.5)-TiO_2_ sample an additional band is seen at 3360 cm^−1^: the band is tentatively ascribed to the presence of another kind of OH species [59], induced by the presence of Fe at the NPs surface, as the same band was hardly discernible with the Fe(1.0)-TiO_2_ sample, but still visible with the Fe(3.5)-TiO_2_ sample, in agreement with the, respectively, low and high Fe contents. Outgassing at 150 and 300 °C (spectra not shown) led to progressive surface dehydroxylation and desorption of weakly held (monodentate) carbonate species, without relevant differences among the studied samples. As a whole, IR spectroscopy confirms that the presence of Fe induces changes in the NPs surface, especially in the OH stretching region.

**Figure 6:**
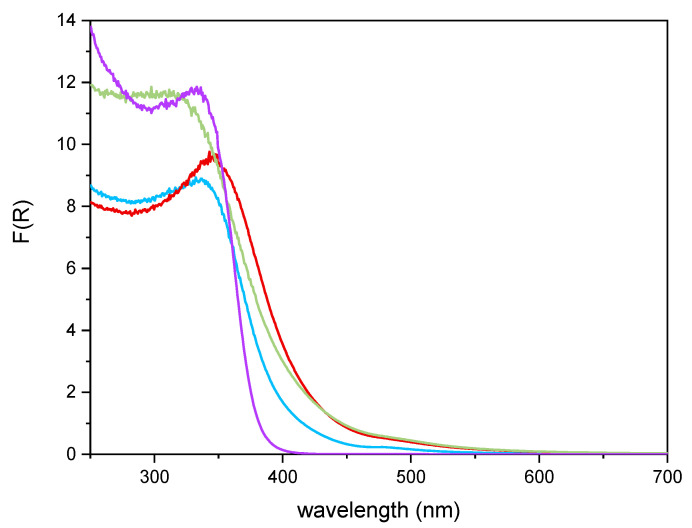
DR-UV-Vis spectra obtained with the undoped TiO_2_ sample (purple line), Fe(3.5)-TiO_2_ (green line), Fe(2.5)-TiO_2_ (red line), Fe(1.0)-TiO_2_ (light blue line).

**Figure 7:**
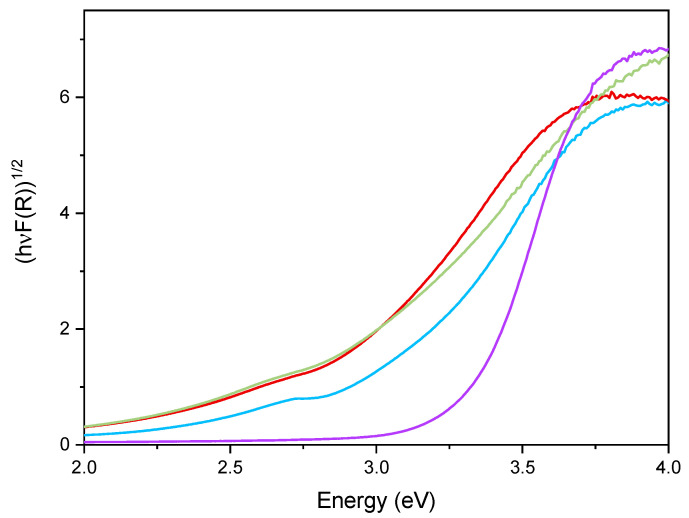
Tauc’s plot applied to the undoped TiO_2_ sample (purple line), Fe(3.5)-TiO_2_ (green line), Fe(2.5)-TiO_2_ (red line), and Fe(1.0)-TiO_2_ (light blue line).

Figure 6 reports the DR UV–Vis spectra of the powder samples. The undoped TiO_2_ sample showed absorption below 400 nm, due to the O_2_^−^ to Ti^4+^ Charge Transfers (CT) transitions. Upon Fe addition, different onsets of absorption (i.e., at longer wavelength) are observed depending on overall Fe content. Additionally, with Fe-doped samples, a new signal is observed at 475 nm, ascribed in the literature to d–d transition of octahedrally coordinated Fe^3+^ ions, likely located at the particles surface, as already observed with other types of samples obtained by impregnation of TiO_2_ [60]. Such Fe^3+^ species belong to Fe_2_O_3_ clusters or Fe-oxo/hydroxide clusters (too low and/or too poorly crystalline to be detected by XRD), likely located at the particles surface [61,62]. The presence of such signal complicates the evaluation of the band gap in the Fe-doped samples. Therefore, the corresponding band gap energy (Eg) values reported in Table 2 were calculated by applying three different methods, namely, the linear extrapolation of the spectra absorption edge (Figure 6), the Tauc’s plot method (Figure 7), for indirect semi-conductors (F(R)xhν)^1/2^ in agreement with to the presence of anatase for the whole set of samples) and a literature method that requires subtracting the dopant contribution from the Tauc’s plot [50]. Regardless which the method is applied, Eg values are obtained by a graphical manipulation of the original DR UV Vis spectra: for the sake of completeness, the average values of the Eg obtained by using the three methods are also reported in Table 2.

As a whole, the Eg value was observed to decrease by increasing the Fe content, especially up to a nominal content of 2.5 wt.%, proving that Fe doping was also effective in the bulk and did not concern only the particles surface; however, at the highest Fe content the effect on Eg was smaller, in agreement with the results of XPS analysis, showing the occurrence of an Fe-enriched surface.

### 3.2. Photocatalytic Activity Results

Experimental tests were carried out using 1.5 g L^−1^ for the three Fe(x)-TiO_2_ samples and undoped TiO_2_ (where x is the weight percentage of Fe) and considering a solution containing 10 mg L^−1^ of AO7 dye. Table 3 reports the list of the used photocatalysts and the amount of adsorbed AO7 normalized to the photocatalyst mass (g).

From the analysis of the photocatalytic tests results, it was possible to infer that the optimal amount of doping was equal to 2.5 wt. % Fe, because a higher decrease of AO7 relative concentration was obtained with Fe(2.5)-TiO_2_ photocatalyst than the Fe(1.0)-TiO_2_ and Fe(3.5)-TiO_2_ samples.

**Figure 8:**
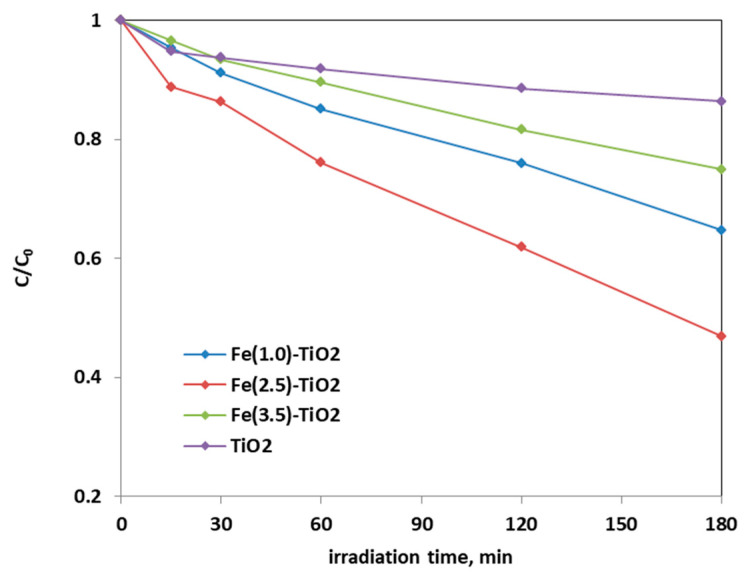
Photocatalytic discoloration of AO7 aqueous solution under visible irradiation as obtained with Fe(1.0)-TiO_2_ (light blue), Fe(2.5)-TiO_2_ (red), Fe(3.5)-TiO_2_ (green), and undoped TiO_2_ (purple) photocatalysts.

**Figure 9:**
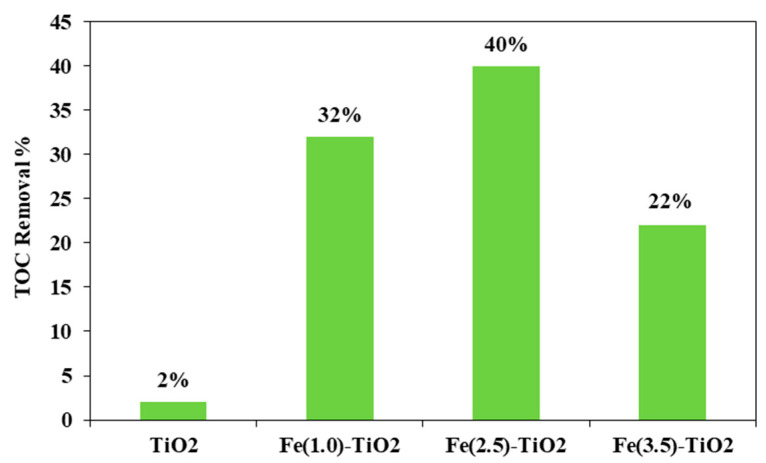
TOC Removal (%) from the AO7 aqueous solution after 180 min of visible using Fe(1.0)-TiO_2_, Fe(2.5)-TiO_2_, Fe(3.5)-TiO_2_, and undoped TiO_2_ photocatalysts.

Figure 8 shows the photocatalytic discoloration process of the three Fe-doped photocatalysts, in comparison with (less active) undoped TiO_2_, highlighting the optimal iron amount: after 180 min irradiation, the Fe(2.5)-TiO_2_ photocatalyst has led to a color removal of approximately 53%. The photocatalyst with the lowest amount of iron (1.0 wt. %) evidenced a discoloration efficiency of 35%, while at the highest iron amount (3.5 wt. %), the photocatalytic performance worsened. Indeed, the low values of discoloration found for undoped mesoporous TiO_2_ exclude the possible sensitization by the AO7 dye. Additionally, the AO7 relative concentration did not decrease in presence of visible light and in the absence of photocatalyst, indicating a negligible effect of photolysis [28]. Figure 9 shows the mineralization efficiency (TOC removal %) of the undoped TiO_2_ and the Fe(1.0)-TiO_2_, Fe(2.5)-TiO_2_, and Fe(3.5)-TiO_2_ photocatalysts.

The TOC removal analysis evidenced that with the Fe(2.5)-TiO_2_ photocatalyst the highest mineralization efficiency, equal to ~40%, was obtained after 180 min of visible illumination. Lower values of TOC removal were found for the other iron-doped photocatalysts. In particular, 32% and 22% of TOC removal were observed for Fe(1.0)-TiO_2_ and Fe(3.5)-TiO_2_ samples, respectively. However, the mineralization efficiency for the undoped TiO_2_ was substantially lower and equal to about 2%. The higher photocatalytic performances of the Fe(x)-TiO_2_ photocatalysts with respect to the undoped TiO_2_ could be associated to the partial replacement of Ti^4+^ ions by Fe^3+^ ions, promoting the charge separation of photogenerated electrons and holes as electron acceptors and reducing the recombination phenomena of electron–hole pairs, as reported in the literature [63]. However, to understand the behavior as a function of iron content, the dark adsorption values are considered for the different prepared photocatalysts (Table 3). It can be seen that the specific amount of adsorbed AO7 was 0.09, 0.17, 2.08, and 5.70 mg g^−1^ for the undoped TiO_2_, Fe(1.0)-TiO_2_, Fe(2.5)-TiO_2_, and Fe(3.5)-TiO_2_, respectively. Despite the lower value of band gap of the sample Fe(3.5)-TiO_2_ (Table 2), no higher activity was observed under visible light with respect to Fe(2.5)-TiO_2_. Such a result could be due to the strong adsorption of AO7 found during the dark equilibration phase on the sample Fe(3.5)-TiO_2_, which appeared strongly colored orange also. Nevertheless, this photocatalysts is less active. It is possible to argue that the high amount of AO7 coordinated to the surface prevented the photocatalyst surface being hit by the photons. Indeed, the main emission of the white LEDs [64] partially fits the absorption spectra in visible range of the target dye [51], so the latter could be responsible of the shield of the radiation, which becomes unable to reach the photocatalyst surface. The values of the isoelectric point can play a role regarding this behavior, controlling the adsorption of pollutant and photocatalytic activity [30]. The addition of Fe to the mesoporous TiO_2_ at increasing amounts leads to a shift of pH_IEP_ towards high pH values, also modifying the surface as pointed out in the characterization section. The anionic dye AO7 can interact with the protonated groups on the photocatalysts surface through the sulfonate negative group. As the pH_IEP_ value is the higher for Fe(3.5)-TiO_2_, the amount of positive charges will be higher at the spontaneous pH of the AO7 solution in comparison with the others samples, inducing a favorable adsorption. However, the presence of a surface enrichment of iron species as found by XPS, could be an additional cause, as well as the fact that higher dopant levels favor the formation of defects which, in turn, favor the electron-hole recombination.

**Figure 10:**
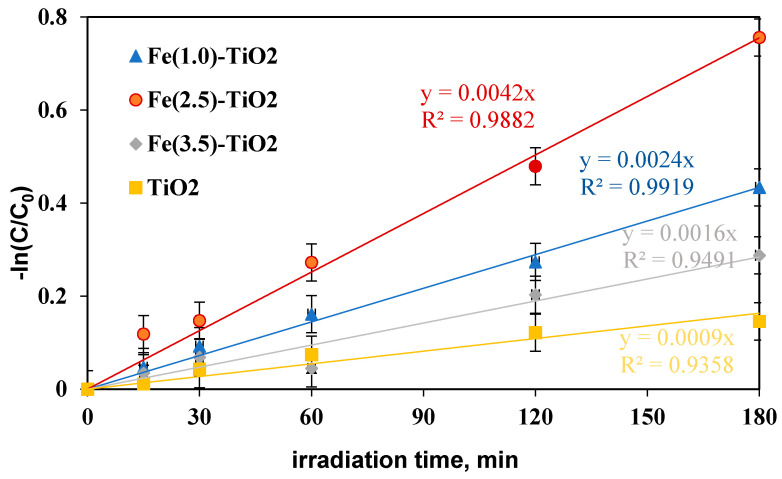
−ln(C/C_0_) versus irradiation time (min) for evaluation of discoloration kinetic after 180 min of visible irradiation using undoped TiO_2_ (yellow), Fe(1.0)-TiO_2_ ( blue), Fe(2.5)-TiO_2_ (red), and Fe(3.5)-TiO_2_ (gray) photocatalysts.

To define the Fe(2.5)-TiO_2_ sample as the optimized photocatalyst towards the photodegradation of the AO7 dye, it was necessary to analyze the discoloration and mineralization kinetics. It must be considered that the typical kinetic mathematical model applied for photocatalytic reactions is that of Langmuir-Hinshelwood [65] and for low initial concentration of pollutants the expression can be reduced to a pseudo-first-order kinetics and therefore the apparent first order kinetic constants can be determined by reporting the integrated form of kinetics considering the mass balance in a batch reactor [28]. The kinetic constant values (k_dec_ for discoloration and k_min_ for mineralization) were evaluated in Figure 10 reporting –ln(C/C_0_) versus time of irradiation for undoped TiO_2_ and for the Fe(1.0)-TiO_2_, Fe(2.5)-TiO_2_, and Fe(3.5)-TiO_2_ photocatalysts, showing that the pseudo-first-order kinetics well fits the discoloration obtained curves.

The four kinetic constants obtained for the discoloration of AO7 varied in the following order:

k_decFe(2.5)-TiO_2__ > k_decFe(1.0)-TiO_2__ > k_decFe(3.5)-TiO_2__ > k_decTiO_2,__ indicating the fast discoloration rate of the AO7 solution and therefore the highest photocatalytic activity was found for the Fe(2.5)-TiO_2_ sample.

In this study, a photocatalyst load equal to 1.5 gL^−1^ was initially applied, the highest discoloration efficiency having been reached with Fe(2.5)-TiO_2_. However, literature reports much lower discoloration efficiency (20%) using Fe-doped TiO_2_ [66] with an AO7 initial concentration of 20 mg L^−1^ and a photocatalyst dosage equal to 1 g L^−1^. Furthermore, the kinetic constant was also significantly lower (k_dec_ about equal to 0.0016 min^−1^) after 180 min of visible irradiation, while in this study using 1.5 g L^−1^ of Fe(2.5)-TiO_2_ and AO7 initial concentration of 10 mg L^−1^ a k_dec_ about 2.6 times higher was found [66].

**Figure 11:**
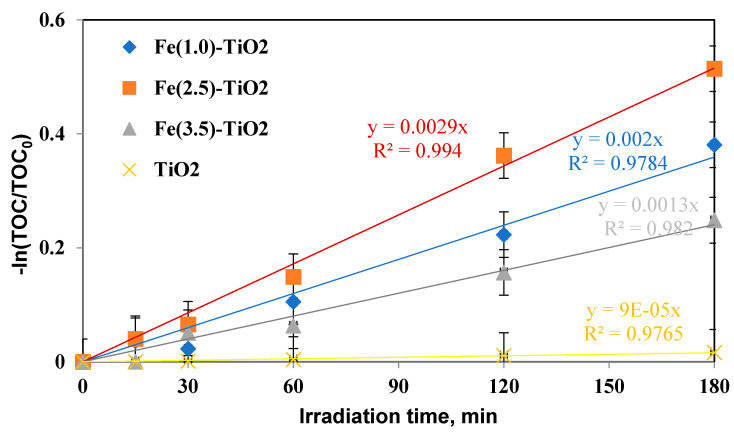
−ln(TOC/TOC_0_) versus irradiation time (min) for evaluation of mineralization kinetic after 180 min of visible irradiation using undoped TiO_2_ (yellow), Fe(1.0)-TiO_2_ ( blue), Fe(2.5)-TiO_2_ (orange), and Fe(3.5)-TiO_2_ (gray) photocatalysts.

The mineralization kinetic constants were similarly evaluated (Figure 11) and again showed an increase in the following order:

k_minFe(2.5)-TiO_2__ > k_minFe(1.0)-TiO_2__ > k_minFe(3.5)-TiO_2__ > k_minTiO_2__, consequently confirming that the mineralization of AO7 with Fe(2.5)-TiO_2_ was also faster than that with the Fe(1.0)-TiO_2_, Fe(3.5)-TiO_2_ photocatalysts and with respect to undoped TiO_2_.

From the results on the discoloration efficiency, mineralization efficiency, and the respective kinetics obtained with the three iron-doped TiO_2_ and with the undoped TiO_2_, it was possible to affirm that the photocatalyst which mostly contributes to the photocatalytic degradation process of the AO7 solution was the Fe(2.5)-TiO_2_ sample.

**Figure 12:**
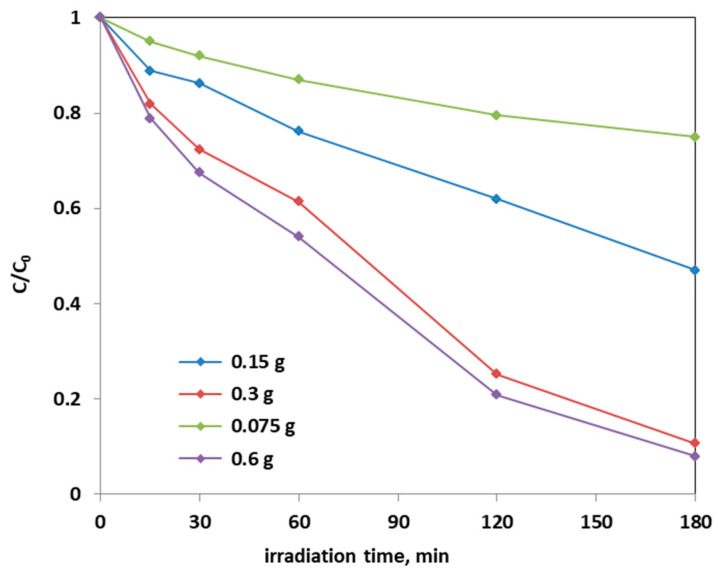
Photocatalytic discoloration of AO7 with different loads of the optimized Fe(2.5)-TiO_2_ sample under visible light irradiation.

Further photocatalytic tests were carried out to establish the optimal dosage of Fe(2.5)-TiO_2_ photocatalyst, which showed the higher photodegradation activity of AO7 aqueous solution: in Figure 12, it is possible to observe the decrease in the relative concentration of AO7 as a function of the Fe(2.5)-TiO_2_ photocatalyst dosage.

**Figure 13:**
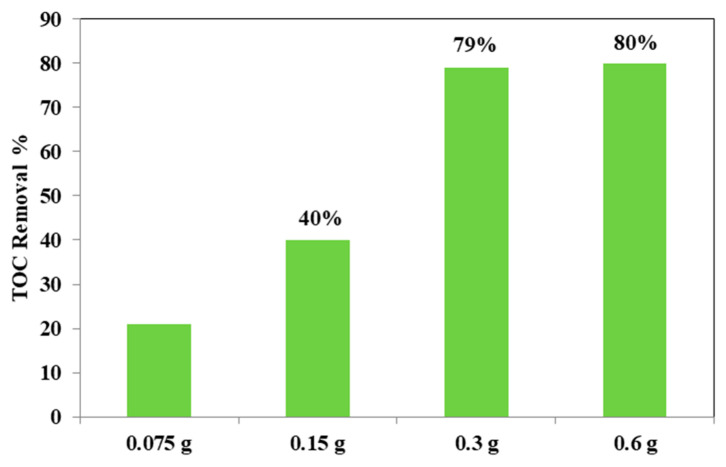
TOC removal (%) from the AO7 aqueous solution after 180 min of visible irradiation using different loads of optimized Fe(2.5)-TiO_2_ photocatalyst.

From the Figure 12, it could be considered that at higher photocatalyst load, there was an increase of the discoloration efficiency of the AO7 solution. In fact, the photocatalytic test with the lowest amount (0.75 g L^−1^) of optimized Fe(2.5)-TiO_2_ photocatalyst resulted in ~20% efficiency of the color removal; the one with the 1.5 g L^−1^ dose of Fe(2.5)-TiO_2_ led to ~45% discoloration efficiency, while the test with 3 g L^−1^ of Fe(2.5)-TiO_2_ showed an efficiency of approximately 90%, slightly less than that obtained by using 0.6 g photocatalyst in 100 mL solution. It has been observed that, although with 0.6 g optimized photocatalyst the decrease of relative concentration is higher, it was more advantageous to use 0.3 g Fe(2.5)-TiO_2_ photocatalyst, because using twice the amount of photocatalyst would imply further operating costs with an almost negligible increase of the discoloration efficiency. Figure 13 showed that the TOC removal did not vary significantly and was about 80% both with 0.3 g and 0.6 g of Fe(2.5)-TiO_2_ photocatalyst; however, reducing the weight of the photocatalyst resulted in the lower removed TOC.

**Figure 14:**
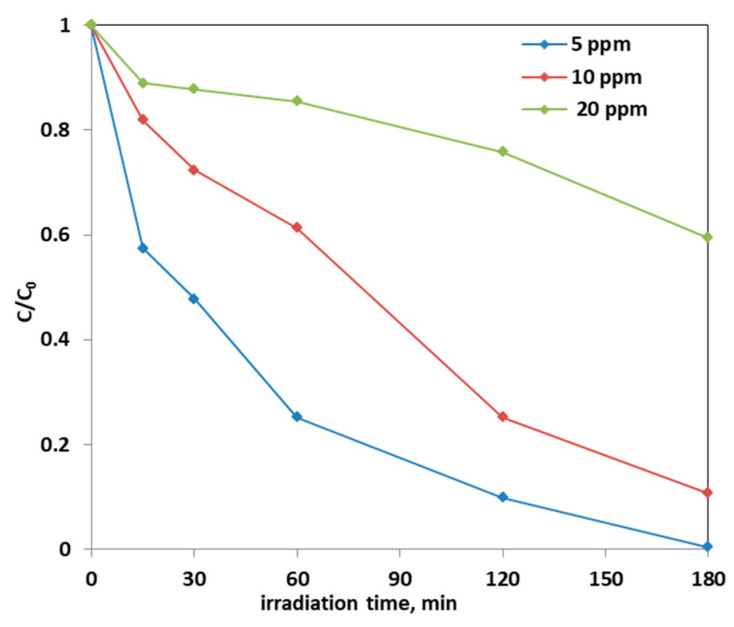
Photocatalytic discoloration of AO7 solution with 0.3 g of optimized Fe(2.5)-TiO_2_ photocatalyst changing the AO7 initial concentration under visible light irradiation.

Note that the highest discoloration kinetics was reported in the literature using a dosage equal to 0.25 g of Fe-doped TiO_2_ catalyst in 100 mL of solution. Precisely, in the article [67] in which the AO7 initial concentration is 25 ppm, the discoloration kinetic constant value was estimated be equal to 0.0016 min^−1^. Instead, in this work, in which the optimal dosage of Fe(2.5)-TiO_2_ catalyst was 0.3 g and the AO7 initial concentration is 10 ppm, the kinetic constant was 0.0119 min^−1^, i.e., one order of magnitude higher. Subsequently, the effect of the initial concentration of AO7 on the photodegradation process was assessed. Figure 14 reports the photocatalytic discoloration process when the initial AO7 concentration was increased from 5 ppm up to 20 ppm using 0.3 g of the optimized photocatalyst (Fe(2.5)-TiO_2_).

**Figure 15:**
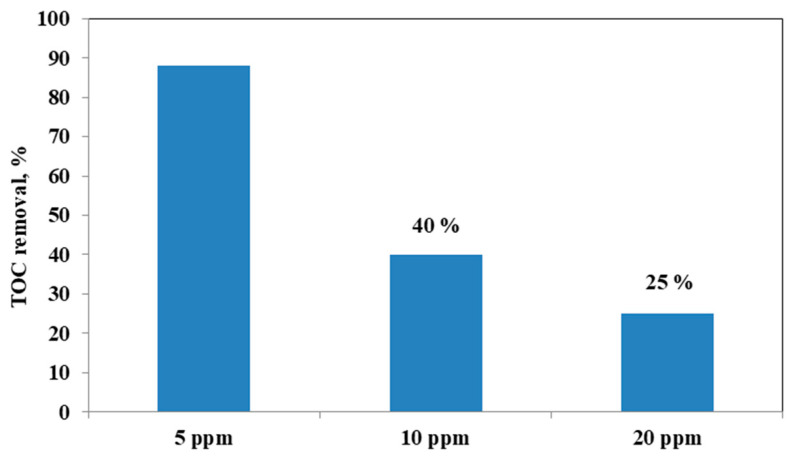
TOC Removal (%) after 180 min of visible light irradiation with 0.3 g of optimized Fe(2.5)-TiO_2_ photocatalyst changing the AO7 initial concentration.

It could be observed that with the increase in the initial AO7 concentration, the decrease in the efficiency of the solution discoloration was obtained. In particular, considering 0.3 g of the same optimized photocatalyst the efficiency of photocatalytic discoloration after 180 min of visible irradiation was equal to 41% with 20 ppm of AO7 initial concentration, while with a lower value of AO7 initial concentration (10 ppm) the efficiency of photocatalytic discoloration increased to 90%. However, the color of the solution was completely removed after 180 min of visible irradiation using an AO7 initial concentration of 5 ppm. This behavior can be explained considering that, with the increase in AO7 initial concentration dye, a higher number of dye molecules are adsorbed on the photocatalyst surface. Therefore, a significant fraction of visible light is absorbed by AO7 itself rather than the photocatalyst particles. As a consequence, the penetration of light to the surface of the photocatalyst decreases [68]. Furthermore, the mineralization efficiency also increased when the initial concentration of AO7 decreased (Figure 15).

**Figure 16:**
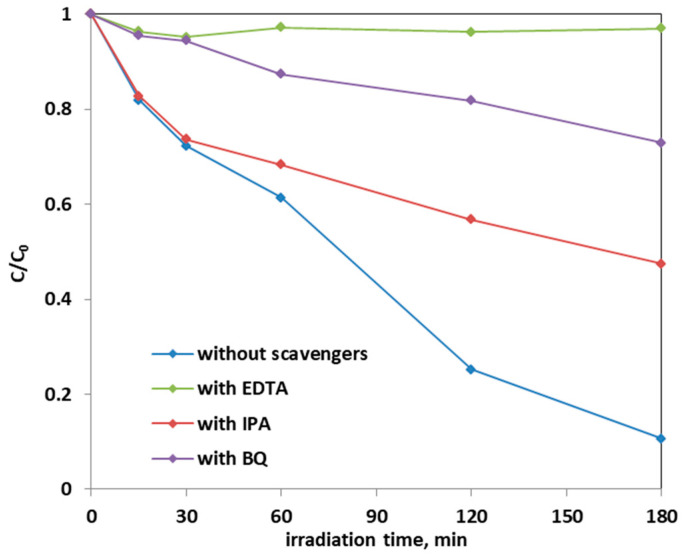
Effect of the scavenger molecules for the degradation of AO7 aqueous solution using 0.3 g of optimized Fe(2.5)-TiO_2_ photocatalyst under visible-light irradiation.

The effect of scavenger molecules on the aqueous solution was evaluated to understand the role that the oxygen reactive species (ROS), such as hydroxyl radicals, positive holes, and superoxide ions, had in the photocatalytic AO7 discoloration mechanism. In particular, isopropanol (IPA), benzoquinone (BQ), and ethylenediaminetetraacetic acid sodium (EDTA), which captures hydroxyl radicals, superoxide ions, and positive holes h^+^, respectively, were added to the AO7 solution. Figure 16 shows the results on the discoloration process using 0.3 g of the optimized Fe-TiO_2_ photocatalyst with a concentration of the scavenger molecules equal to 10 mmol L^−1^. The presence of these scavenger molecules reduced the discoloration efficiency of the AO7 solution from 90% to a value of 53% in the presence of IPA. The addition of BQ in the solution led to a discoloration efficiency of 27%, while the introduction of EDTA inhibited completely the photocatalytic discoloration process. This result evidences that the main ROS involved in the discoloration mechanism are both superoxide ions and, in particular, positive holes. This result is consistent with literature dealing with photocatalytic degradation of AO7 dye [28,69].

**Figure 17:**
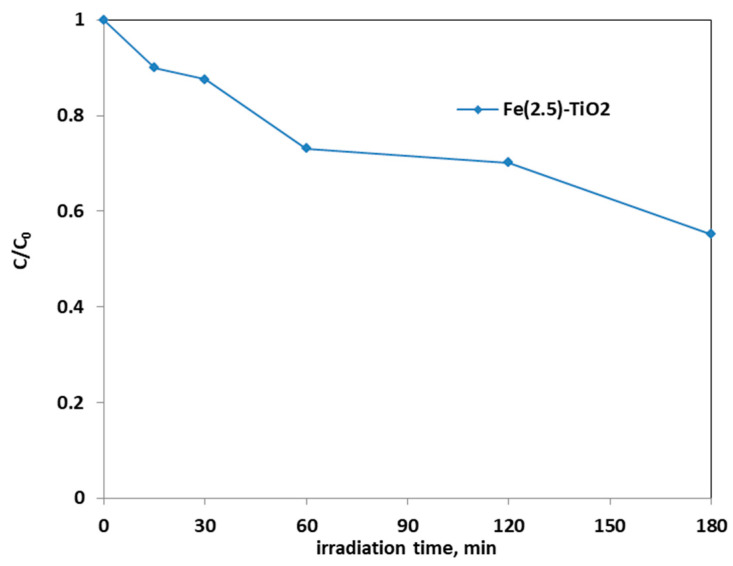
Photocatalytic discoloration of aqueous solution containing phenol (10 mg L^−1^) using 0.3 g of optimized Fe(2.5)-TiO_2_ photocatalyst under visible light irradiation.

Finally, a photocatalytic test with 10 mg L^−1^ of the phenol solution was performed using 0.3 g of the optimized Fe(2.5)-TiO_2_ photocatalyst under visible light irradiation (Figure 17).

It is possible to observe that the photodegradation efficiency of the aqueous solution containing phenol was equal to 45%. This allowed us to affirm that the optimized photocatalyst is able to effectively degrade not only the AO7 dye, but also another colorless and very stable organic compound, such as phenol, confirming also the absence of sensitization phenomena when photocatalytic tests were carried out in presence of AO7.

### 3.3. Possible Reaction Mechanism and Literature Comparison

As the Fe doping does not affect the crystal size and specific surface area (BET) of the samples to a certain extent, the photocatalytic activity of the catalysts is not dependent by these parameters. On the other hand, the presence of the right content of iron in TiO_2_ lattice improves the separation of the photogenerated electrons and holes, inhibiting their recombination.

In particular, it is recognized in the literature [21] that Fe^3+^ ions in the framework of titania capture the charge carriers, electron(e^−^)and holes (*h*^+^), according to the following equations:

Fe^3^^+^ + *h*^+^ = Fe^4^^+^ (4)

Fe^3^^+^ + e^−^ = Fe^2+^ (5)

These reactions could occur sequentially to the photogeneration of charge carriers by the doped semiconductor because the energy level for Fe^3+^/Fe^4+^ oxidation is above the valence band edge of TiO_2_, whereas the energy level for Fe^3+^/Fe^2+^ reduction is below the conduction band edge of the host semiconductor [21].

Moreover, Fe^2+^ and Fe^4+^ ions are less stable with respect to Fe^3+^ ions; therefore, the trapped charges can be released giving Fe^3+^ ions and could migrate to the surface promoting the photocatalytic degradation of the target pollutant.

Further reactions of Fe^2+^ ions are the transformation to Fe^3+^ ions by transferring electrons to adsorbed O_2_ on the surface of TiO_2_ (Equation (6)) or to Ti^4+^ ions (Equation (7)). Then, the generation of reactive species such as O_2_^.−^ and OH• could occur by reactions (8) and (9).

Fe^2^^+^ + O_2_(ads) → Fe^3^^+^ + O_2_^.−^ (6)

Fe^2^^+^ + Ti^4^^+^ → Fe^3^^+^ + Ti^3^^+^ (7)

Ti^3^^+^ + O_2_(ads) → Ti^4^^+^ + O_2_^.−^ (8)

Fe^4^^+^ + OH^−^(ads) → Fe^3^^+^ + OH•(ads) (9)

Degradation products could be obtained by the interaction of the pollutant with the reactive oxygen species according to Equations (10)–(12):

pollutant (ads) +O_2_^.−^ → degradation products (10)

pollutant (ads) + OH•(ads) → degradation products (11)

pollutant (ads) + *h*^+^ → degradation products (12)

The latter equation (Equation (12) seems to be the higher responsible for the photocatalytic degradations of the pollutants, as demonstrated by the photocatalytic tests in the presence of the scavenger molecules.

The photocatalytic activity of Fe-(2.5)-TiO_2_ was compared with iron-doped TiO_2_ materials available in the literature for the degradation of several organic compounds including AO7 (Table 4).

The data reported in Table 4 evidenced the higher performances of the Fe-doped TiO_2_ studied in this work with respect to doped samples synthesized by hydrothermal method [35]. In particular, the Fe-doped TiO_2_ of this work showed an AO7 photodegradation efficiency equal to about 90% after a shorter irradiation time than Fe-doped TiO_2_ samples [42,67]. The other Fe-TiO_2_ formulations reported in literature achieved a higher photocatalytic degradation efficiency but at longer treatment time [21,70].

## 4. Conclusions

Three Fe-doped mesoporous TiO_2_ photocatalysts (with 1, 2.5, and 3.5 wt. % Fe nominal content) were prepared by a modified template assisted sol–gel method, using Ti-*n*-butoxide as TiO_2_ precursor, in the presence of iron(III) chloride hexahydrate as Fe precursor and of the tri-block copolymer Pluronic 123 as template.

Like the undoped mesoporous TiO_2_, all the Fe-doped samples were 100% anatase, as no crystalline Fe_x_O_y_ phases were detected by XRD, in agreement with the low Fe content, although DR UV–Vis spectra showed the presence of Fe oxo/hydroxide species, likely occurring as clusters located at the nanoparticles surface, in agreement with ζ-potential measurements and XPS analysis.

The band gap of the samples was found to decrease in the presence of Fe, indicating the occurrence of actual Fe doping, whereas XPS analysis showed the existence of an Fe-enriched TiO_2_ surface, likely due to the synthesis method, which allows a partial introduction of Fe in the TiO_2_ bulk and a simultaneous Fe dispersion at the TiO_2_ surface.

The Fe-doped photocatalysts showed enhanced performances in the photodiscoloration of AO7, accomplished by its mineralization under visible light irradiation, in contrast with the negligible photoactivity evidenced by the (undoped) TiO_2_.

The sample at 2.5 wt. % Fe (Fe-(2.5)-TiO_2_) showed the best AO7 photoremoval efficiency, ascribed to the lower band gap value with respect to undoped TiO_2_ and favorable adsorption properties towards the dye. On the other hand, a higher Fe content (3.5 wt. % Fe) was detrimental to the photocatalytic activity, probably because the iron species localized at the surface acted as recombination sites for photogenerated charges.

On the best photocatalyst (Fe-(2.5)-TiO_2_), kinetics was explored, resulting in fair agreement with a pseudo-first order behavior. The optimal conditions were found with a dosage of 3 g/L, 10 ppm of AO7 initial concentration, leading to 90% of photodiscoloration after 180 min of visible irradiation.

The main active species in the photocatalysis were investigated using scavenger molecules in the photoreaction medium, indicating that the positive holes, and in turn the superoxide radical ions are mainly involved.

For the sake of completeness, results obtained in this work with the best photocatalyst (Fe-(2.5)-TiO_2_) and the optimized operative conditions were compared to those reported in literature for similar Fe-doped TiO_2_ photocatalysts: it was shown that the adopted preparation method leads to material with superior performance and that a shortened time of irradiation is required to reach a high photoconversion.

Finally, the photodegradation of a phenol solution was studied in the presence of Fe-(2.5)-TiO_2_, showing its ability to remove up to 45% of such a recalcitrant molecule after 180 min under visible light, evidencing the absence of photosensitization phenomena when the azo-dye was used as model pollutant.

## Figures and Tables

**Table 1:** Reaction conditions.

#	Values
White LEDs strip power	10 W
Light intensity	13 mW·cm^−2^
Volumetric air flow rate	150 cm^3^·min^−1^
Total volume of AO7 solution	100 mL

**Table 2:** Some relevant structural and textural properties of the studied samples as obtained by X-ray powder Diffraction (A = anatase) and N_2_ isotherms at −196 °C. Band gap energy values, as obtained from DR UV–Vis spectra by applying different methods suggested by literature. Values of pH_IEP_ (pH at the isoelectric point), as obtained by ζ-potential measurement.

Sample	Crystallite Size (nm) ^a^	QPA Results (wt.%) ^b^	pH_IEP_	SSA (m^2^ g^−1^) ^c^	Band Gap Energy (Eg, eV) ^d,e,f^	Nominal Fe/Ti Atomic Ratio
				Total Pore Volume (cm^3^ g^−1^)	Average Value of Eg	XPS Surface Fe/Ti Atomic Ratio
TiO_2_	10.0 ± 0.6 (A)	100 (A)	2.36	150	3.28, ^d^ 3.35,^e^ 3.32 ^f^	0
				0.28	3.31	0
Fe(1.0)-TiO_2_	9.4 ± 0.4 (A)	100 (A)		135	3.10, ^d^ 3.01 ^e^, 3.08 ^f^	0.014
			4.14	0.25	3.06	0.034
Fe(2.5)-TiO_2_	8.4 ± 0.6 (A)	100 (A)		130	2.88, ^d^ 2.74 ^e^, 3.03 ^f^	0.037
			4.58	0.25	2.88	0.069
Fe(3.5)-TiO_2_	7.1 ± 0.6 (A)	100 (A)		145	2.99, ^d^ 2.70 ^e^, 3.02 ^f^	0.052
			5.08	0.24	2.90	0.128

^a^ As obtained by applying the Williamson–Hall method. ^b^ As obtained by Rietveld refinement. ^c^ As obtained by applying the BET method. ^d^ As obtained by linear extrapolation of the DR UV–Vis spectra absorption edge. ^e^ As obtained by applying the Tauc’s plot method for indirect band gap semiconductor (F(R)*hυ)^1/2^. ^f^ As obtained by applying the method reported in [50].

**Table 3:** AO7 adsorbed with respect to the load of photocatalyst.

Sample	$\frac{mg_{AO7\;\mathrm{absorbed}}}{g_{\mathrm{catalyst}}}$
TiO_2_	0.09
Fe(1.0)-TiO_2_	0.17
Fe(2.5)-TiO_2_	2.08
Fe(3.5)-TiO_2_	5.70

**Table 4:** Comparison on the photocatalytic activity of the Fe(2.5) -TiO_2_ sample under visible light irradiation with the available literature.

Catalyst	Irradiation Time	Contaminant	Photocatalytic Degradation Efficiency (%)	Reference
Fe-TiO_2_	300 min of visible light (0.05 *mol. %* Fe)	4-nitrophenol	~92% (10 mgL^−1^)	[70]
Fe-TiO_2_	480 min of visible light (0.09% *wt*/*wt* FeCl_3_)	Yellow XRG dye	~37% (100 mg L^−1^)	[35]
Fe-TiO_2_	360 min of visible light (0.2 wt. % Fe)	Methyl orange	~72% (20 mg L^−1^)	[21]
Fe-TiO_2_	360 min of visible light (2.0 wt. % Fe)	AO7	~53% (35 mg L^−1^)	[42]
Fe(2.5)-TiO_2_	180 min of visible light (2.5wt. % Fe)	AO7	~90% (10 mg L^−1^)	[this paper]

## Data Availability

All data are available from the authors.
